# Combined Lumbar Plexus-Sciatic Nerve Block for Urgent Lower Limb Surgery: A Good Alternative in Patients With High Anesthetic Risk

**DOI:** 10.7759/cureus.58763

**Published:** 2024-04-22

**Authors:** Iliass Ennour idrissi, Manal Rhezali, Abdellah Enourhbi, Taoufik Abou Elhassan, Hicham Nejmi

**Affiliations:** 1 Anesthesiology and Critical Care, Mohammed VI University Hospital, Faculty of Medicine and Pharmacy of Marrakech, Cadi Ayyad University, Marrakech, MAR

**Keywords:** high risk patients, regional anesthesia, sciatic nerve block, lumbar plexus nerve block, peripheral nerve block

## Abstract

The use of peripheral nerve blocks has grown significantly in recent years not only because of the progress and availability of the materials necessary for its realization but also for enhancing patient safety. Anesthesia in the elderly or individuals with high anesthetic risk is always a challenge for the practitioner. Hence, the importance of peripheral nerve blocks in avoiding the side effects associated with general or spinal anesthesia. In this report, we present a case series of five patients with high anesthetic risk (classified American Society of Anesthesiologists (ASA) III or IV) who underwent different lower limb surgeries under a combined lumbar plexus-sciatic nerve block. The goal of this case series was to evaluate the effectiveness of this technique as an anesthetic alternative for these different types of surgical procedures.

## Introduction

The importance of regional anesthesia in lower limb surgery has undergone significant development in recent years, driven by several factors. One reason is the technical advancements in equipment, coupled with the widespread adoption of ultrasonography [1]. Another contributing factor is the steadily increasing proportion of elderly and very elderly patients, often presenting with multiple comorbidities, who require limb surgery [2]. These developments collectively contribute to enhanced patient safety.

Peripheral nerve blocks have emerged as the preferred analgesic or even anesthetic strategy for numerous lower limb surgeries, either alone or in combination with peri-medullary or general anesthesia. Additionally, they offer effective postoperative analgesia, thus reducing the need for opioid use. Among these techniques, the lumbar plexus block stands out as a delicate yet highly effective method for both analgesic and anesthetic purposes, particularly in hip and knee surgery [3].

This report aims to assess the efficacy of the combined lumbar plexus-sciatic nerve block (CLP-SNB) in various surgical indications involving the lower limb, while also evaluating its tolerance in particularly fragile patients classified as American Society of Anesthesiologists (ASA) III or IV, depending on the case.

## Case presentation

Peripheral nerve block techniques

Lumbar Plexus Block (LPB)

**Figure 1:**
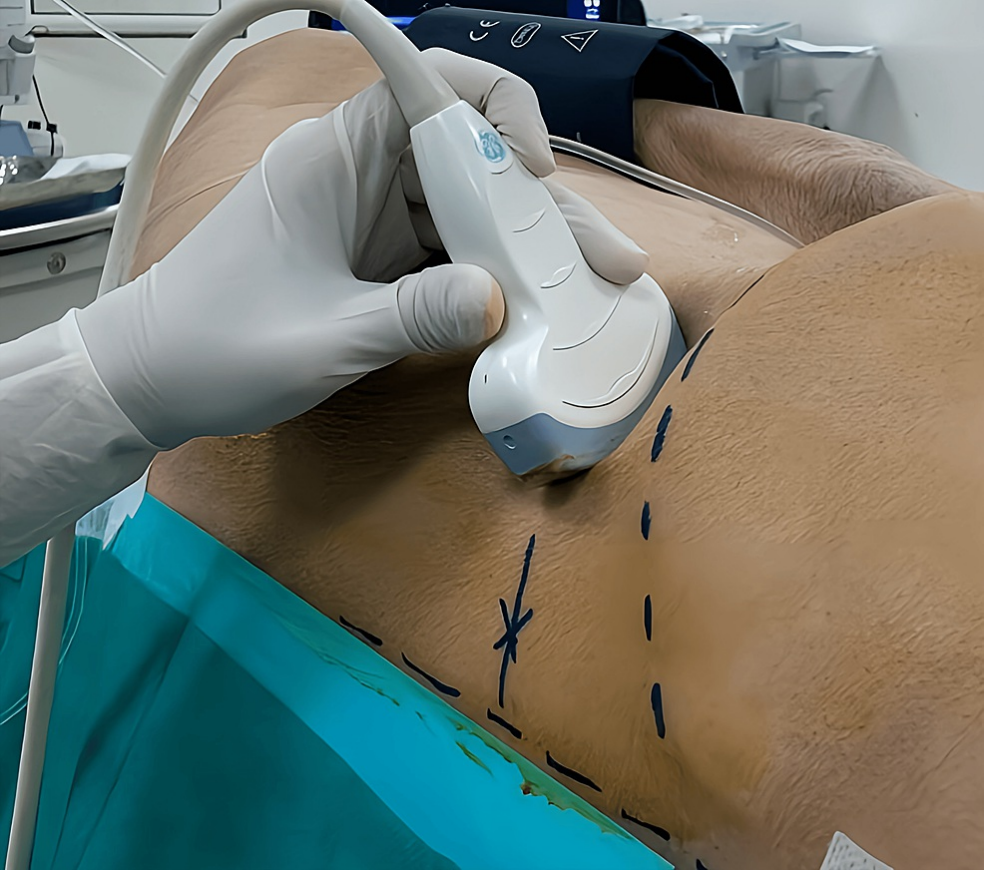
Probe Placement for Lumbar Plexus Visualization "X" marks the needle insertion point

The LPB was performed utilizing Shamrock's sub-costal approach, employing an ultrasound-guided technique in conjunction with a neurostimulator. The patient was positioned in lateral decubitus on the healthy side, and a low-frequency curved probe was utilized for visualization. The probe was positioned subcostally and oriented towards the L4 vertebra (Figure 1), enabling visualization of the vertebral body and the transverse process of L4, which are surrounded by three muscles: the erector spinae posteriorly, the quadratus lumborum above the transverse process, and the psoas muscle anteriorly. The lumbar plexus is visualized within the iliopsoas muscle mass.

A 100 mm-long short-bevel needle connected to a neurostimulator was inserted along a line drawn parallel to the orientation of the probe passing through L4, approximately 4 cm above the line of the spinous processes. Stimulation intensity, frequency, and duration were set at 2 mA, 1 Hz, and 0.1 ms, respectively.

Sciatic Nerve Block (SNB)

The SNB was performed in the subgluteal space with the patient in the same position, offering the advantage of patient immobility and the ability to block the posterior cutaneous nerve of the thigh effectively. The same probe was utilized, positioned along a line extending from the greater trochanter to the ischial tuberosity. Within this trajectory, the quadratus femoris muscle is visualized in the deep plane, with the gluteus maximus muscle appearing more superficially. Between these two muscles lies a hyperechoic structure: the sciatic nerve.

Case 1

A 49-year-old male adult with a medical history notable for hypertension, dilated cardiomyopathy characterized by severe biventricular dysfunction, and a reduced left ventricular ejection fraction (LVEF) of 27%, as well as valvular disease including moderate mitral and aortic regurgitations, along with severe tricuspid regurgitation, was classified as ASA physical status class IV.

The patient presented to the Emergency Room (ER) with a posterior thigh abscess necessitating surgical drainage. He underwent surgery under CLP-SNB. The duration of the anesthetic procedure was 30 minutes due to difficulties encountered during the LPB, which required two attempts. The surgical procedure lasted 40 minutes without any hemodynamic compromise or adverse events. Additionally, no associated sedation was required during the procedure.

Case 2

A 65-year-old female with a medical history including type 2 diabetes mellitus for over 11 years managed with insulin, dyslipidemia, and obesity, was diagnosed with dilated hypokinetic cardiomyopathy characterized by severe biventricular dysfunction and an LVEF of 25%.

She presented to the ER with gangrene affecting both feet, necessitating mid-leg amputation of both limbs. The procedures were performed 48 hours apart for each limb. Our primary objective was to ensure optimal cardiac and hemodynamic tolerance with minimal invasiveness and swift recovery.

On both occasions, the combined nerve block was successfully achieved without the need for sedation. However, during the second surgery, the patient experienced arterial hypotension, necessitating the administration of vasopressors. It remains unclear whether this event was directly related to the nerve block or attributed to the multifactorial nature of her heart failure.

Regrettably, despite the successful surgeries, the patient passed away five days after the second procedure due to cardiac decompensation and multiple organ failure.

Case 3

A 62-year-old male, with a medical history notable for chronic obstructive pulmonary disease (COPD) at stage III on the mMRC (Modified Medical Research Council) Dyspnoea Scale, a lifelong smoking habit, hypertension, and obliterating arteriopathy of the lower limbs, presented to the ER with dry gangrene affecting the right lower limb secondary to neglected chronic limb ischemia, necessitating an above knee amputation (AKA). This patient was classified as ASA class IV.

The anesthetic procedure was successfully completed on the first attempt and lasted 24 minutes, facilitating a 70-minute surgery. Following the procedure, the patient experienced hypotension due to bleeding, which was corrected by the transfusion of one unit of red blood cells.

Case 4

An 80-year-old male, with a medical history notable for arterial hypertension, type 2 diabetes mellitus, and chronic renal failure, presented with a surgical site infection necessitating the removal of osteosynthesis material. His past surgical history includes the surgical treatment of a hip fracture using a gamma nail 13 weeks prior to admission. This patient was classified as ASA class III.

The combined nerve block was successfully administered in 20 minutes, achieving adequate sensory and motor blocks. However, despite the successful nerve block, the patient remained anxious and required the administration of 3 mg of midazolam for supplementary sedation to facilitate the completion of the surgical procedure. The surgery lasted 55 minutes without any hemodynamic compromise

Case 5 

A 70-year-old male, with a medical history notable for complicated pulmonary tuberculosis resulting in chronic respiratory failure (stage II on the mMRC Dyspnoea Scale) and extensive lung destruction evident on chest radiography, classified as ASA III, presented with a patellar fracture following a domestic accident. The planned surgical intervention involved a patellar tension band wiring.

Surgery was postponed for 48 hours to allow for the assessment and optimization of pulmonary comorbidities before proceeding with the procedure. However, in consideration of preserving respiratory function, we opted for the utilization of a CLP-SNB. The procedure was carried out successfully, with the satisfaction of both the patient and the surgeon.

The following table summarizes all the patients and surgical characteristics, as well as the procedure timings and success.

**Table 1:** Surgical Characteristics and Procedure Timings for Patient Cases M: male; F: female

Patients	Age (years)	Gender	Number of Comorbidities	Diagnosis	Surgical Procedure	Duration of anesthetic procedure (minutes)	Duration of surgery (minutes)	Associated sedation
CASE 1	49	M	3	Posterior thigh abscess	Surgical drainage	30	40	No
CASE 2	65	F	4	Right foot ischemia	Mid-leg amputation	26	50	No
CASE 2	65	F	4	Left foot ischemia	Mid-leg amputation	25	50	No
CASE 3	62	M	3	Gangrene right leg	Mid-thigh amputation	24	70	No
CASE 4	80	M	4	Infected osteosynthesis (long gamma nail)	Removal of material	20	55	Yes
CASE 5	70	M	5	Patella fracture	Patellar tension band wiring	30	70	No

## Discussion

Nowadays, anesthesiologists and surgeons are confronted with an increasing number of elderly patients with multiple co-morbidities, who present with various surgical indications. Surgery on the lower limbs, particularly in post-traumatic cases but not exclusively, represents a significant portion of these indications. However, literature on anesthesia techniques employing combined peripheral nerve blocks for lower limb surgeries is limited [4-6].

The utility of performing surgery under peripheral nerve blocks is particularly pertinent for elderly, frail individuals, and/or those with significant comorbidities, with an ASA score ≥ 3 [4]. This is especially crucial in urgent surgeries that cannot be delayed, even if underlying comorbidities are unstable, unassessed, and unoptimized.

In such extreme situations, where functional reserves are low and quickly destabilized, the risks and benefits of each anesthesia technique must be carefully weighed. Pharmacokinetic and pharmacological changes induced by anesthetic agents during general anesthesia can lead to severe hemodynamic consequences, while spinal anesthesia may cause a profound reduction in cardiac output and blood pressure due to sympathetic blockade [7].

Peripheral nerve blocks offer advantages such as improved hemodynamic stability, prolonged postoperative analgesia, and reduced incidence of thromboembolic events. Among various regional anesthesia techniques, the CLP-SNB is the method that appears to be the most versatile in terms of effectiveness regardless of the surgical indication. This is due to the sensitive and motor innervation of the lower limb, which dermatomes, myotomes, and sclerotomes are theoretically all anatomically covered by this combination [8].

Previous studies have often assessed the combination of lumbar plexus block with subgluteal sciatic nerve block for similar surgical indications or as an alternative in high-risk patients [9-11]. Our study aimed to combine both techniques, recommending them for high ASA score patients requiring emergency lower limb surgery, regardless of the surgical indication.

Overall, the combination of plexus block and sciatic nerve block proved effective, both for simple procedures like patellar bracing and more complex and painful surgeries like AKAs.

## Conclusions

The aging of the population increases the proportion of patients with multiple comorbidities, constituting a real challenge for their perioperative care. The necessity to develop innovative techniques for enhancing hemodynamic stability is crucial for improving outcomes. These techniques offer distinct advantages, including improved hemodynamic stability, prolonged postoperative analgesia, and a reduced risk of thromboembolic events. In this context, the CLP-SNB could emerge as a credible, safe, and effective alternative. The implications of this case series suggest extending the application of CLP-SNB to a broader patient population and a wider array of surgical procedures.
